# A qualitative longitudinal study of motivation in the REtirement in ACTion (REACT) physical activity intervention for older adults with mobility limitations

**DOI:** 10.1186/s12966-023-01434-0

**Published:** 2023-04-26

**Authors:** Rosina Cross, Colin Greaves, Janet Withall, Marlene Kritz, Afroditi Stathi

**Affiliations:** 1 Department for Health, University of Bath, Claverton Down, BA2 7AY Bath, UK; 2 College of Medicine and Health, University of Exeter, St Luke’s Campus, Heavitree Road, EX1 2LU Exeter, UK; 3 School of Sport, Exercise and Rehabilitation Sciences, University of Birmingham, Edgbaston, B15 2TT Birmingham, UK; 4 Curtin School of Population Health, Curtin University, Kent St, WA 6102 Bentley, Australia

**Keywords:** Exercise, Ageing, Randomised Controlled Trial, Motivation, Group, Maintenance

## Abstract

**Background:**

Physical activity (PA) is beneficial for older adults’ health, however they remain the least active age group in the UK. This qualitative longitudinal study aims to understand motivations in older adults receiving the REACT physical activity intervention, through the lens of self-determination theory.

**Methods:**

Participants were older adults randomised to the intervention arm of the Retirement in ACTion (REACT) Study, a group-based physical activity and behaviour maintenance intervention to prevent decline of physical functioning in older adults (≥ 65 years). Stratified purposive sampling by physical functioning (Short Physical Performance Battery scores) and 3-month attendance was employed. Fifty-one semi-structured interviews were conducted at 6, 12 and 24-months with twenty-nine older adults (Mean age (baseline) = 77.9 years, SD 6.86, 69% female) and at 24-months with twelve session leaders and two service managers. Interviews were audio recorded, transcribed verbatim and analysed using Framework Analysis.

**Results:**

Perceptions of autonomy, competence and relatedness were associated with adherence to the REACT programme and maintenance of an active lifestyle. Motivational processes and participants’ support needs, changed during the 12-month REACT intervention and across the 12-months post-intervention. Group interactions were an important source of motivation during the first six months but increased competence and mobility drove motivation at the later stages (12 months) and post-intervention (24 months).

**Conclusions:**

Motivational support needs vary in different stages of a 12-month group-based programme (adoption and adherence) and post-intervention (long-term maintenance). Strategies to accommodate those needs include, (a) making exercise social and enjoyable, (b) understanding participants’ capabilities and tailoring the programme accordingly, (c) capitalising on group support to motivate participants to try other activities and prepare sustainable active living plans.

**Trial registration:**

The REACT study was a pragmatic multi-centre, two-arm, single-blind, parallel-group, RCT (ISRCTN registration number 45627165).

**Supplementary Information:**

The online version contains supplementary material available at 10.1186/s12966-023-01434-0.

## Background

Physical activity (PA) is key to the maintenance of physical [1–9], cognitive [10–12], mental [13–15] and social health [16] in later life. However, there is an age-related decline in PA in England, with 31% of 65-to74-year-olds reporting < 30 min of moderate-to-vigorous PA per week, rising to 53% of people aged ≥ 75 years [17, 18]. Consequently we, need to understand what motivates older adults to increase and maintain activity levels.

Self-determination theory (SDT) proposes that motivation that fulfils fundamental human needs, determines behaviour [19]. Systematic reviews of SDT-based PA interventions demonstrates that autonomous rather than controlled motivation is linked to the adoption and maintenance of PA behaviours [20]. SDT suggests that autonomous motivation is determined by the extent to which needs for autonomy, competence and relatedness are met [19, 21]. Autonomy support involves giving an individual a choice of how to engage in a behaviour [22–25]. Competence support involves helping an individual feel successful and confident at the behaviour [19, 26–28], and relatedness support provides an individual with opportunities for behaviour-supporting social interaction [19, 22, 25]. To date, SDT- based PA interventions have primarily relied on quantitative methodologies for evaluating behaviour change processes [20, 29–31].

Qualitative research increases our understanding of an intervention’s mechanisms of behaviour change [27, 32–36] and factors impacting intervention effectiveness [33, 37], implementation and acceptability [27, 38, 39]. Existing qualitative PA research is predominantly cross-sectional, providing little information on how motivational processes change over time [32, 35]. Longitudinal qualitative research (LQR), defined as the collection of data at multiple time points is sparse [37, 40–42] but can address this gap in the literature by providing insight as to how motivational processes and need-satisfaction may change during an intervention [36, 37, 41].

One value of longitudinal qualitative research has been to determine how motivation may be internalised and its impact on behavioural maintenance [41, 42]. Individuals with diabetes who participated in a 12-week walking programme described extrinsic factors (i.e., commitment and obligation) as dominant when initiating PA behaviour (baseline), whilst autonomy, competence and enjoyment were more important for behavioural maintenance at 12-weeks and 12-months post-intervention [42].

Motivators of behaviour change are likely to change over the life course. A range of studies highlight that older adults differ from younger adults in how they engage with PA interventions [33–35, 43–45]. This gap in the literature highlights the need for qualitative longitudinal research examining the processes of long-term PA behaviour change in older adults.

Our qualitative longitudinal study explored motivations for PA and ongoing engagement among participants in a 12-month PA and behavioural maintenance programme. The perspectives of session leaders and service managers delivering the programme were also explored. Through the lens of SDT, the study provides an in-depth evaluation of the impact of the proposed mechanisms of action of the intervention in the context of a large-scale, multi-site trial.

## Methods

### Study design

Semi-structured interviews were conducted with participants receiving and service providers delivering a PA intervention, as part of the REACT randomised controlled trial (RCT). Interviews were conducted at six (mid-intervention), 12 (post-intervention) and 24-months (longer-term follow up). Ethical approval was provided by the National Health Service (NHS) Southeast Coast–Surrey Research Ethics Committee (15/LO/2082).

The REACT study was a pragmatic multi-centre, two-arm, single-blind, parallel-group, RCT (ISRCTN registration number 45627165) [46–49]. The REACT study assessed the effectiveness and cost-effectiveness of a community-based, multimodal, group PA programme focusing on improving mobility, strength and balance in older adults (> 65 years) with impaired mobility [47]. The REACT intervention included social and educational components designed to support PA behaviour maintenance. Results at 24-months showed that physical functioning (measured using the Short Physical Performance Battery) was significantly higher in the intervention group than in the control group (adjusted mean difference of 0·49 [95% CI 0·06–0·92]) [47]. Further, the REACT programme was cost-effective [47].

**Fig. 1:**
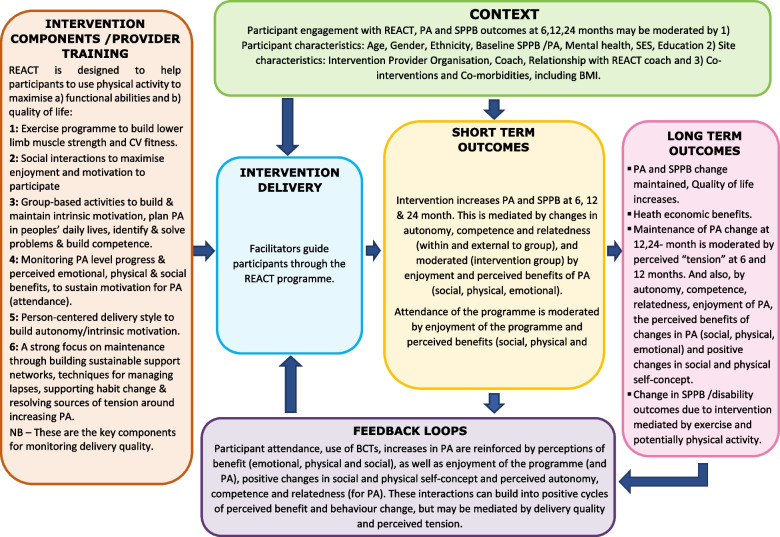
REACT logic model

Group-exercise sessions were delivered over 12-months, with twice weekly exercise sessions for the first 12-weeks, then weekly sessions up to 52-weeks [48, 50]. Health behaviour maintenance sessions were delivered weekly from weeks 9-to-24, then monthly from weeks 28-to-52. These sessions included behaviour change techniques (BCTs) and processes which drew on SDT to support key psychological needs (autonomy, competence and relatedness) and to enhance motivation for PA [19, 51]. These psychological processes are illustrated (along with other proposed intervention mechanisms) in the REACT Logic Model (Fig. 1), which shows how the intervention’s proposed mechanisms of change were intended to motivate and sustain changes in PA [48, 50].

### Sampling and recruitment

A stratified purposive sampling strategy was employed to sample participants from the REACT intervention arm and ensure rich and diverse experiences. Participants were stratified and assigned to one of four groups according to their attendance at group exercise sessions and their physical functioning at baseline (Table 1) [52]. Physical functioning was assessed with the Short Physical Performance Battery test (SPPB) which measures normal walking speed over 4 m, time to complete five repeated rises from a chair, and completion of three standing balance tasks of increasing difficulty. Each measure was scored from 0 (inability to complete the test) to 4 (best performance) and the sum of the three component scores was calculated (0–12). Other characteristics such as age, sex, ethnicity, intervention group, intervention session leader and provider were also considered.

**Table 1:** Interview sampling matrix

Group	Session Leader	SPPB (High 8–9, Low 4–7)	3-Month Attendance High (≥ 50%)		3-Month Attendance Low (< 50%)	Recruitment Target per group
Group 1	F1	High SPPB		1	1	4
		Low SPPB		1	1	
Group 3	F1	High SPPB		1	1	4
		Low SPPB		1	1	
Group 4	F3	High SPPB		1	1	4
		Low SPPB		1	1	
Group 5	F4	High SPPB		1	1	4
		Low SPPB		1	1	
Group 6	F5	High SPPB		1	1	4
		Low SPPB		1	1	

For sampling, attendance was classified as high when attending at least 50% of group-exercise sessions, and low when attending less than 50%. SPPB scores were categorised as frail or prefrail. Scores of 4–7 were considered frail/Low SPPB and scores from 8 to 9 considered pre-frail/High SPPB (frailty-classification recommendations of the European Medicines Agency) [53].

All session leaders involved in the delivery of the REACT programme were invited for interviews after the completion of the 12-month group programmes they were leading. Informed written consent was obtained from participants during recruitment to the REACT study (Additional file 1) [50].

### Data collection

Semi-structured topic guides were developed and piloted with the REACT service user advisory group (Additional file 2). The participant topic guides were designed to explore experiences of the REACT programme: reasons for engagement, perceived benefits, barriers and enablers for REACT attendance and daily PA and behaviour change processes (e.g. motivation). These are illustrated in the REACT logic model (Fig. 1). The longitudinal study design was key to exploring experiences in detail at major transition points in the REACT programme [36]. Topic guide questions were designed to encourageparticipants to reflect on past experiences in conjunction with present perspectives [54, 55]. The session leaders’ topic guide was developed to understand their experiences of delivering REACT, their perspectives on participant motivational processes and the programme’s strengths and points for refinement.

### Data collection procedures and informed consent

Participants who had expressed an interest in the qualitative study (via the recruitment and consent process for the REACT trial) were contacted via telephone and asked to participate in three semi-structured interviews at each time point. The semi-structured, face-to-face interviews were conducted either in participants’ homes or in community centres. At 24-months, all interviews were conducted at participants’ homes. Session leaders took part in individual interviews at 12-months. Interviews were conducted by two experienced PhD interviewers, RC (PhD) and JdK (PhD).

Interviews were recorded using an Olympus VN-741PC password-protected encrypted digital recorder, on average lasting 48-minutes (range = 22–89 min). All interview data were immediately transferred to a University of Bath owned password-protected and encrypted server, before deletion from the digital recorder. Interviews were transcribed verbatim and de-identified.

### Data analysis

Framework analysis was used to analyse the interviews [56], using the NVivo qualitative research software (NVivo 12) to organise the data. A predetermined framework consisting of the three SDT components (relatedness, autonomy, competence) was employed as an initial coding frame. The analysis sought to identify individual narratives and within-person processes of change, as well as to draw out common themes [57]. Transcripts were coded by RC, AS and SW, with regular discussions about emergent themes. Emergent themes were compared and contrasted with the theorised processes of change identified by SDT [39]. Factors influencing participant motivation and engagement in PA at 12-and-24-months were explored and linked to participants’ responses at six-months. Data collection at multiple time-points allowed a longitudinal, qualitative evaluation of participants’ experiences, and motivations for the adoption and maintenance of active lifestyles at three time points.

### Trustworthiness, rigour and transparency

The lead researcher (RC) familiarised herself with the context of the study via conversations with participants prior to the interview, and observations of the delivery of the REACT programme. Paraphrasing participant responses to ensure correct interpretations during the interviews was used to increase credibility and minimise bias [58, 59]. Rigour was enhanced through (a) researcher reflection on interview notes and emerging themes; (b) the development of a transcription protocol; (c) the employment of multiple coders. Transparency was ensured via a detailed audit trail and extensive discussion of emerging themes between coders. The COnsolidated criteria for REporting Qualitative research (COREQ) Checklist was used to ensure the study is reported comprehensively [58–60] (Additional file 3).

## Results

### Participant characteristics

Using the interview sampling matrix (Table 1), we purposively selected four participants, one from each stratum, from five intervention groups at three intervention sites (Bath/Bristol, Devon and Birmingham).

#### Participants

At 6-months, 12 women (71%) and five men (29%) were interviewed (Table 2). Baseline age ranged from 68 to 89 years. SPPB (47% frail; 53% pre-frail), and attendance (47% low attendance; 53% high attendance) were relatively balanced.

**Table 2:** Summarises participant characteristics and illustrates baseline and 24-month SPPB scores and programme attendance

Pseudonym	Group	Gender	Age (baseline)	SPPB (baseline)	SPPB 24 months	Attendance	Interview 6-Month	Interview 12-Month	Interview 24-Month
Dorothy	1	F	68	7	12	72%	×	×	×
Cordelia	1	F	88	4	5	27%	×		
Etta	1	F	70	8	10	98%	×	×	×
Anandi	1	F	76	9	9	49%	×	×	×
Darsha	1	F	77	9	8	81%		×	×
Iris	2	F	89	9	8	79%	×	×	×
Mary	2	F	76	4	4	35%	×		
Cecil	2	M	69	4	12	65%	×		
Frederick	2	M	87	8	9	90%	×	×	×
Geraldine	3	F	78	8	10	61%	×	×	×
Arman	3	M	71	8	9	95%	×	×	×
Valerie	3	F	86	8	9	61%		×	×
Rita	3	F	76	8	10	75%		×	×
Beatrice	4	F	80	9	9	96%	×	×	
Alvita	4	F	76	5	8	91%	×	×	
Arthur	4	M	76	8	9	65%	×	×	
Roger	4	M	84	5	2	87%	×	×	
Ann	5	F	74	7	6	78%	×	×	
Evelyn	5	F	69	7	5	35%	×		
Flora	5	F	79	8	5	30%	×		
John	4	M	83	6	1	56%			×
Sam	2	M	88	5	2	51%			×
Betty	4	F	88	7	7	98%			×
Timothy	6	M	70	9	7	5%			×
Angelina	6	F	73	9	9	65%			×
Mark	6	M	73	9	9	63%			×
Jane	2	F	84	8	10	35%			×
Rachel	4	F	83	6	11	82%			×
Eleanor	6	F	68	8	12	30%			×

At 12-months, it was not possible to reach four women and one man from the 6-month cohort, therefore three additional participants were recruited. The sample comprised of 11 women (73%) and four men (27%), ranging from 69 to 90 years. They were predominantly pre-frail (73%), with high attendance (60%). At 24-months, 13 women (68%) and six men (32%) ranging from 70 to 91 years were interviewed. Most participants were pre-frail (79%) with high attendance (63%) (Table 2).

#### Session leaders

Twelve of the 15 REACT session leaders agreed to participate in semi-structured interviews (4 Bath/Bristol, 4 Birmingham, 4 Devon). Additionally, one research team member who had delivered some of the health behaviour maintenance sessions with three groups (Devon), and two service managers from one provider organization (Bath/Bristol) were also interviewed. The total number of interviewees was 15 (7 male /8 female).

### Findings

**Fig. 2:**
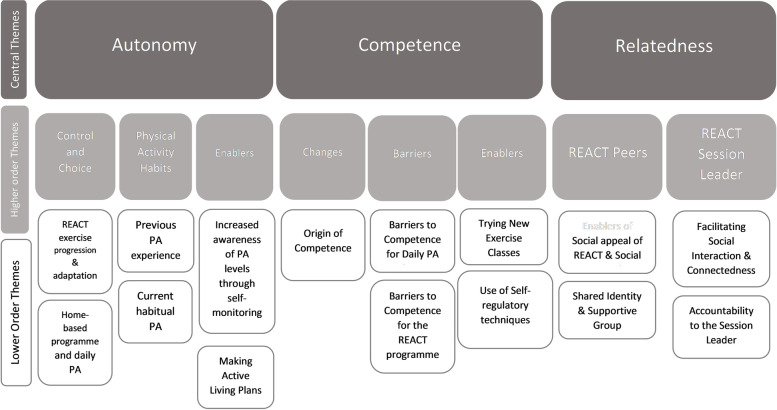
Hierarchy of themes

Findings supported the three pre-determined central themes of Autonomy, Competence and Relatedness, presented in Fig. 2. These included eight higher order themes (HOT)and 14 lower order themes (LOT). The following sections present the similarities and differences in these themes at 6-, 12-and-24-month time points.

**Fig. 3:**
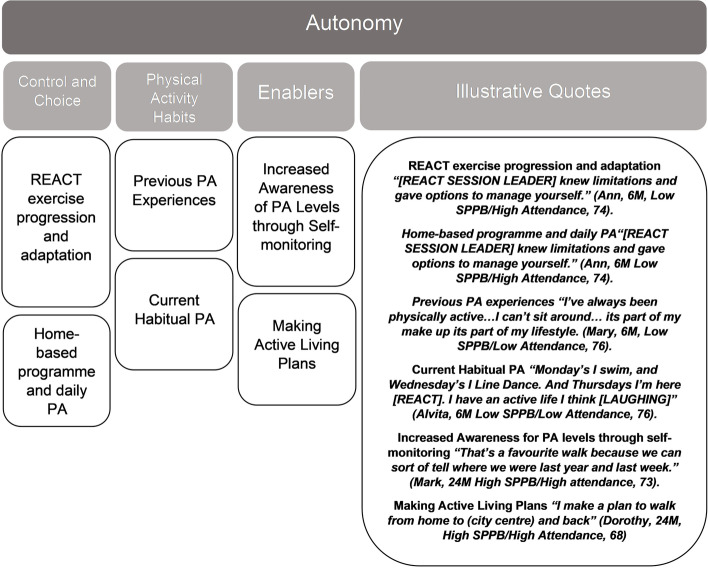
Autonomy

The central theme ‘Autonomy’ was comprised of three higher order themes: Control and choice, Physical activity habits and Enablers illustrated with participant quotes (Fig. 3) and described below.

### Control and choice (higher order theme)

#### REACT exercise progression and adaptation (lower order theme)

Some individuals reported that the freedom to adapt exercise to their individual needs and exerting control over their participation and progression in exercise sessions was key to feeling confident and motivated to engage in and adhere to the REACT programme. Session leaders supported this by (a) knowing participants’ physical limitations, (b) being flexible, (c) adapting exercises to account for mobility limitations and (d) offering tailored choices to individuals. Session leaders confirmed this, highlighting the importance of flexibility when working with a group with a range of capabilities and needs. The need for control over the exercise programme was less commonly reported at 12-months, whereas the need for control of daily activity, external to the programme, post-intervention was reported more often.

#### Home-based programme and daily physical activity (lower order theme)

Participants reported that incorporating PA in daily life was determined by enjoyment and their ability to adapt exercise to their physical abilities. At six-month interviews, participants reported that when the REACT sessions were reduced from two per week to once per week, the choices and the new opportunities for activity that session leaders provided were viewed positively. At 12-and-24-months participants highlighted the importance of being able to adapt REACT exercises to their home environment and incorporate them in their plans to maintain PA.

### Physical activity habits (higher order theme)

#### Previous physical activity experiences (lower order theme)

Most participants spoke about the importance PA had hadheld throughout their lives. They had formed PA habits around activities that incorporated enjoyment and choice. These habits evolved as participants aged to account for physical limitations and included activities such as; walking, gardening, house chores and scheduling exercise around daily routine.

#### Current habitual physical activity (lower order theme)

Participants viewed their current activity as being habitual, focusing on enjoyment and choice. REACT classes became more habitual from six-to-12-months and at 12-and-24-months participants began reporting the inclusion of REACT exercise at home as part of daily activity.

### Enablers (higher order theme)

#### Increased awareness of physical activity levels through self-monitoring (lower order theme)

Participants reported a greater sense of awareness of their PA levels due to self-monitoring, taught during REACT. This awareness was less prominent at six-months, however towards the end of the intervention (12-months) and post-REACT, participants reported that self-monitoring facilitated a sense of control over their daily PA and motivated them to do more.

#### Making active living plans (lower order theme)

As with previous PA experiences, participants highlighted the importance of enjoyment and choice when it came to making future PA plans. Making plans and goal-setting were more common at 12-and-24-months. This is potentially due to completion of the REACT programme, encouragement from REACT session leaders and an increased sense of importance of an active lifestyle. For some participants, goal-setting was not easy to adopt, preferring to exercise when they could, rather than creating additional pressures by goal-setting and committing to action plans. Alternatively, other participants recognised the importance of goal-setting and action-planning to achieve their PA targets. Some participants suggested that goal-setting is a personality trait, while others reported that they had learned to set goals during the REACT programme.

#### Competence

**Fig. 4:**
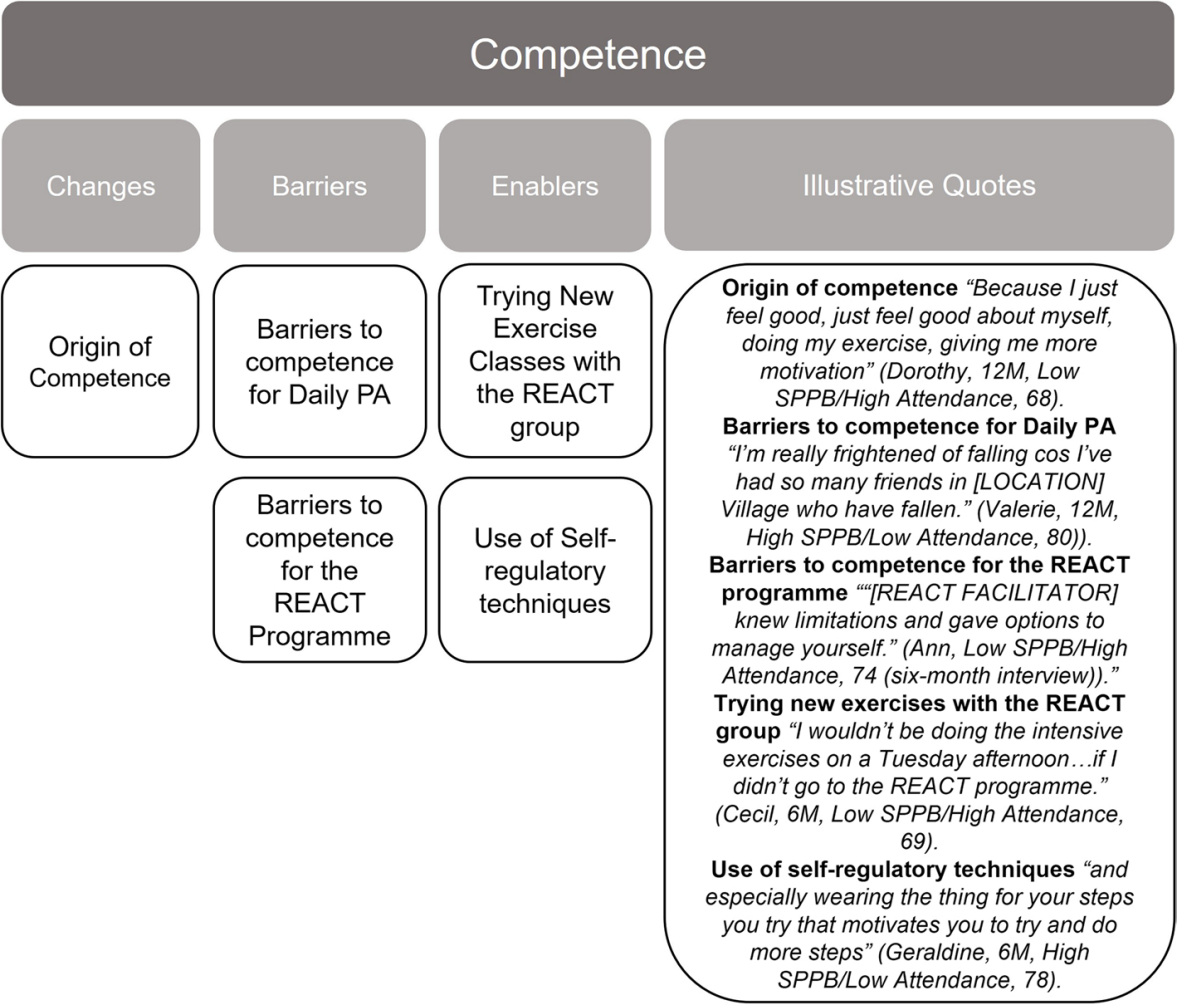
Competence

The central theme ‘Competence’ was comprised of three higher orderthemes; Changes, Barriers and Enablers, illustrated with participant quotes (Fig. 4) and described below.

### Changes (higher order theme)

#### Origin of competence (lower order theme)

Participants reported improvements in competence for PA. This shifted from a cautious reporting of improved competence at six-months to a more enthusiastic declaration of competence at 12-months. At six-months, much of the competence was derived from the REACT session leader’s style of instruction or through the vicarious experiences provided by watching others in the class (social models). Participants reported that gradual progression of exercises and successful execution improved their competence and subsequently their motivation for engagement with REACT and daily PA. At 12-months, participants described deriving competence from their own ability to perform the REACT exercises. Perceptions of competence motivated participants to engage in exercise, independently from the REACT programme. Participants reported that positive physiological and emotional changes further facilitated their perceived competence. Some participants reported that their competence for REACT exercises translated to increased confidence in their ability to replicate these exercises at home. Similarly, the origin of motivation for adhering to REACT and for daily PA transitioned from externally-derived (REACT peers and session leaders) at six-months to becoming more internalised at 12-and-24-months and deriving from their own sense of improved competence.

### Barriers (higher order theme)

#### Barriers to competence /confidence for daily physical activity (lower order theme)

Participants reported barriers to their perceived competence for incorporating PA into their daily lifestyle but reported no barriers to attending REACT. Low competence at six-months stemmed from age-related deterioration and fear of falling, resulting in being cautious about the amount and type of activity they engaged in independent of REACT. However, this was not reported as a barrier to participation in REACT. Low competence was less frequently reported at 12-months. It was mainly triggered by observation of peers who had experienced falls and subsequent loss of mobility.

#### Barriers to competence for the REACT Programme (lower order theme)

Barriers that reduced competence for daily PA at six-and-12-months were not reported as impacting competence for the REACT programme. Enjoyment of REACT, support from session leaders and peers enabled them to overcome potential barriers to attendance.

### Enablers (higher order theme)

Self-regulatory techniques and trying new exercise classes were key enablers of competence.

#### Trying new exercise classes with the REACT Group (lower order theme)

Participants credited REACT as a gateway to participation in exercise classes independent of REACT. Session leaders gave REACT participants the opportunity to sample new exercise classes with the REACT group, leading to some participants joining these classes. Participants and session leaders reported this made up for the transition from two REACT classes a week to one. Session leaders confirmed that while some participants were keen to try new classes, others needed more support. Despite variation in competence for trying new exercise classes, the safe environment provided by the REACT community compensated for lack of competence in some. Consequent participation in the classes further increased competence for PA independent of REACT.

#### Use of self-regulatory techniques (lower order theme)

While some disliked self-monitoring PA behaviours (14%) at six-and-12-months, others reported that self-monitoring PA with pedometers had improved both their competence and motivation for PA. At 24-months, participants employed self-regulatory techniques including self-monitoring, adjusting PA expectations (e.g. based on health), using problem-solving to overcome specific barriers (e.g. walking indoors to stay active when the weather was poor), habit formation (repeating activities regularly until they became a daily routine) and action-planning (e.g. to manage competing commitments) to stay motivated and maintain activity despite challenges (e.g. competing commitments, declining health and adverse weather).

### Relatedness

**Fig. 5:**
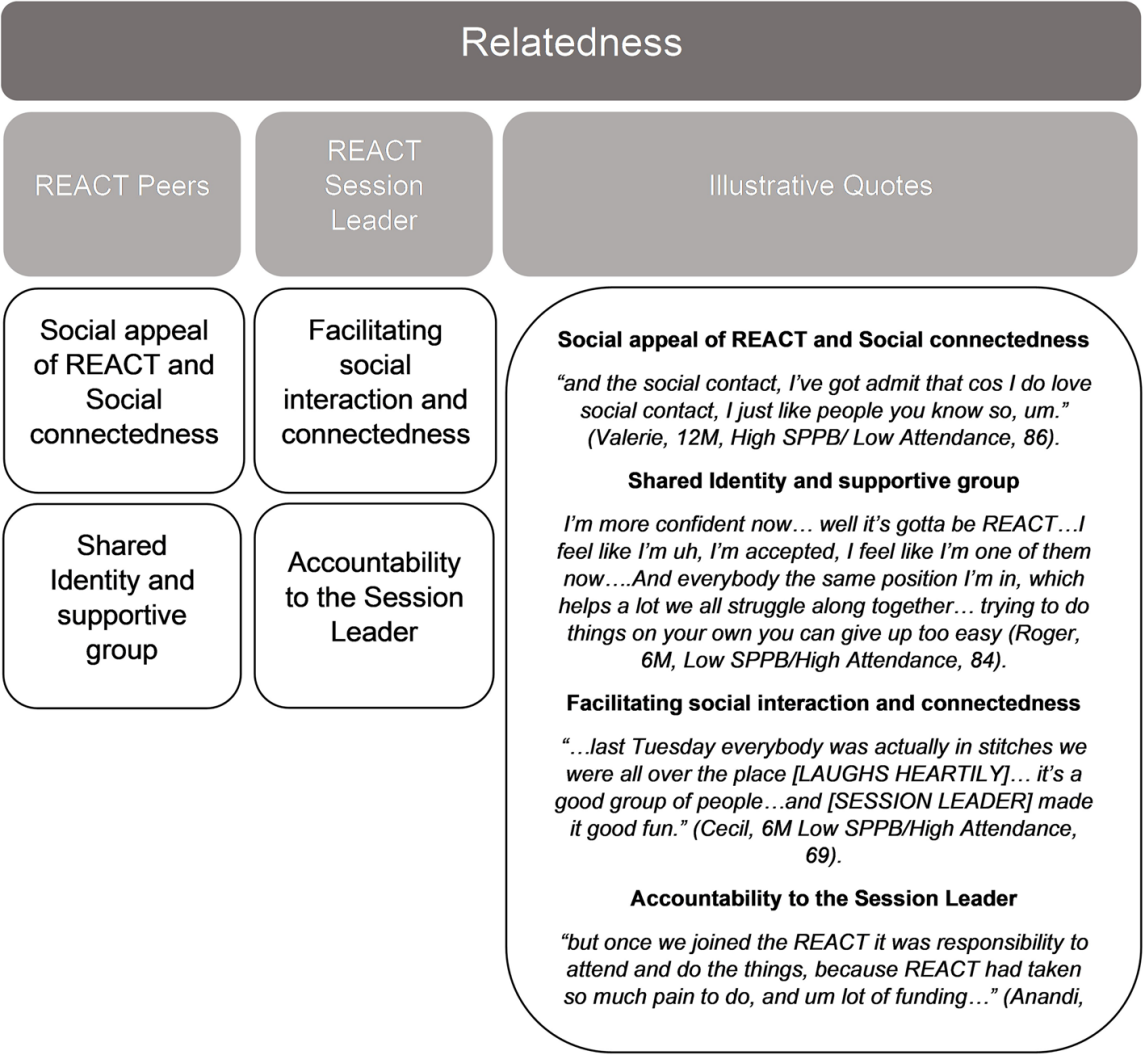
Relatedness

The central theme relatedness was comprised of two higher orderthemes; REACT Peers and REACT Session Leaders, illustrated with participant quotes (Fig. 5) and described below.

### REACT peers (higher order theme)

#### Social appeal of REACT and Social connectedness (lower order theme)

Social appeal of REACT and social connectedness was commonly reported at both six-and-12-months and was characterized by acknowledgement of the importance of social connectedness to the health and wellbeing of older adults. At 12-months there was a shift from simply acknowledging that social connectedness is important for wellbeing, to reporting that social connectedness amongst REACT group members had increased. Increased social connectedness motivated participants to continue engaging with the REACT programme. While social connectedness was important throughout the programme, ways for enhancing social connectedness varied between REACT groups, (i.e. the presence of an ambassador organising social events for one group, or the creation of a carpool to support attendance at another group). Participants reported that feelings of social connectedness enhanced enjoyment of REACT and motivated continued participation in REACT and daily PA.

At 24-months, one year after the completion of the REACT programme, many participants reported enacting independent PA plans. They described having supportive social networks independent of the REACT groups, with their social support needs being met by people independent of REACT. However, after completion of the REACT programme some reported shrinking social connections, highlighting its impact on PA opportunities and physical and mental wellbeing.

#### Supportive Group (lower order themes)

Supportive Group were reported at both six-and-12-month interviews to positively impact both competence and motivation for PA. For some participants, this helped them to overcome barriers. For example, being supported by other members of the group when they were struggling with an exercise, made participants feel they were experiencing a shared challenge.

One REACT group organised a car-pool, to support each other attend REACT. Furthermore, one lady described her REACT group as an opportunity to challenge the perceptions people have of different racial groups. The same group had an ‘ambassador’ that encouraged socialising outside of REACT. Supportive group was less prominent at 24-months with participants sourcing social support independent of REACT.

### REACT session leader (higher order theme)

All participants discussed the importance of REACT session leaders and the impact they had on REACT attendance and daily PA.

#### Facilitating social interaction and connectedness (Lower order theme)

At six-months, participants described REACT session leaders as fostering social interaction amongst the group. They employed games and encouraged PA outside of REACT that was social. This was not reported explicitly at 12-months, however, supportive group was commonly reported at 12-months implying that the fostering of social interaction by the session leaders was more important during the earlier months of the REACT intervention and that, once social connections were made, the group itself became responsible for maintaining them.

#### Accountability to the session leader (lower order theme)

At six-months participants reported a sense of curiosity towards REACT. At 12-months, this transitioned to attaching value to it. Participants reported that REACT session leaders were sources of support and encouragement, especially when experiencing barriers to participation. As such, a sense of accountability to the session leader to attend developed. The development of relationships with REACT session leaders was positive for many, but also had unintended consequences when session leaders changed, new sessions leaders found it difficult to ‘fill the shoes’ of previous session leaders.

## Discussion

This longitudinal qualitative study identified three central themes over the 24-month period, all related to SDT: Autonomy, Competence and Relatedness. Session leaders supported participants in adapting REACT exercise to the home environment and encouraged the adoption of self-regulatory strategies such as self-monitoring, action-planning and problem-solving. Participants reported that having control over their participation in REACT, supported by session leaders, was key to adoption (six-months) and to long-term (12-months) REACT participation. The importance of control here, supports literature highlighting that autonomous rather than controlled motivation is key to behavioural adoption and maintenance [19, 20, 42]. Session leaders further supported autonomy by providing choices and options of REACT exercises. The importance of autonomy support for promoting PA among older adults, aligns with qualitative research emphasising that older adults perceive it important for peer walk leaders to acknowledge the needs of their walking group members and tailor the planned walks to meet members’ capabilities and preferences [33].

Improvements in competence were reported at each timepoint throughout the REACT programme. However, the source of this competence and the subsequent motivation for further PA participation varies across the three time points. At six-months, competence was derived externally from Session Leaders and REACT peers. At 12-and-24-months, competence was derived from personal capability and improvements in physical, social and mental health (i.e. participants attributed improved mobility to increased PA and recognised the importance of active living in maintaining improvements). There was a clear distinction between types of motivation (controlled and autonomous) and internalisation of motivation during REACT. The distinction between types of motivation supports existing literature that extrinsic motivation may be more important in the adoption stage of PA behaviour change with internalisation of motivation being important for sustained behaviour change [19, 21, 31, 37, 41, 42]. Furthermore, this confirms the conceptualisation of motivation within SDT and systematic review evidence showing that intrinsic motivation, is associated with exercise adherence [61].

Participants identified factors commonly cited in the literature as positively affecting competence-related motivation: (a) support to try new classes, (b) breaking down and adapting exercises to suit individual capabilities, (c) use of self-regulatory techniques (self-monitoring and problem-solving) to break down barriers and sustain motivation over time. These self-regulation strategies are reported by several systematic reviews and meta-analyses to be determinants of successful PA interventions [31, 35, 62–66]. Interviews suggested differences in competence at the individual level, that impacted the support needs of some participants, highlighting the importance of addressing individual needs within group context and tailoring support within exercise programmes. Furthermore, perceived physical, mental and social benefits acted in a feedback loop to positively impact competence and motivation for PA (i.e. improved social connectedness with the REACT group motivated continued participation in REACT to maintain this newly formed social network). This feedback loop highlights the importance of experiencing benefits early on in an exercise programme to boost adherence and sustained PA. Barriers to competence were also reported but tended to originate from comparison with peers’ experiences of falls and loss of mobility. This comparison aligns with the concept of vicarious arousal, whereby participants acquire attitudes or emotions towards a phenomenon based on their vicarious experiences of a social model [67]. Negative vicarious experiences can act as inhibitors, undermining competence for a behaviour.

Participants reported a sense of relatedness/social connectedness to both REACT session leaders and their REACT peers. Relatedness was facilitated by exercise that was enjoyable and sociable [35, 63, 68, 69], fostering a shared identity and by a supportive group dynamic [35, 62–66]. At 6-months, participants reported this was important for health and wellbeing and motivating for REACT participation, consistent with SDT [19, 51] literature highlighting the importance of relatedness in PA adoption [25, 37].

Relatedness was equally important at 12-months as it was at six months. Increased relatedness motivated participants to adhere to REACT and maintain relationships among group members, consistent with existing literature highlighting participant relatedness as an important determinant of adherence [27]. Furthermore, supportive groups helped participants overcome barriers to REACT participation (e.g. physical ailments or transport barriers). Social interaction within the groups was perceived as being enjoyable, supporting evidence from systematic reviews and metanalyses that identify both enjoyment [35, 63, 68, 69] and relatedness [35, 62–66] as being key features in PA behaviour change in older adults. At 24-months (one-year post-intervention), social connectedness among REACT group members was, as expected, less prominent and social networks independent of REACT facilitated the support needs of participants.

Session leader support for social interaction and the commitment participants felt to the session leader and REACT peers are commonly cited in the literature as important components of successful active aging programmes [42, 64, 65, 70–72]. In this study we observed a stronger session leader-supported connectedness at the outset of the programme when group cohesion was weak. This was replaced at the later stages of the programme by a stronger group member-supported connectedness.

### Implications for practice

Enjoyment and social connectedness were key motivators for participation. Future interventions should promote enjoyable exercise and facilitate social interaction to foster a sense of relatedness by incorporating ice breaker activities and games into the exercise classes. Autonomy and competence were key factors in older adults’ motivation to participate in REACT. Session leaders delivering exercise programmes for older adults need to support the autonomy and competence of older adults by (a) using a person-centred delivery style (b) focusing on individual progress (c) adapting exercises to suit individual needs.

Some session leaders used transition arrangements to expose participants to exercise opportunities independent of REACT. Transition arrangements, (e.g. providing information and introducing exercise sessions independent of REACT) may be a key strategy to boost competence and allow participants to build a menu of exercise options and avoid creating dependence on one exercise programme. REACT was a research programme with a predefined endpoint, whereas community programmes can be on-going, not requiring transition arrangements. Regardless, programmes should support people to build activity choices and facilitate ways to experience other activities within the organisation or within the community. Research shows that increased competence and relatedness experienced in one programme does not translate to sustained participation in other activities once that programme is gone [73] so capitalising on the positive motivational climate of one programme to support participants to ‘try out’ other activities may be a key strategy for the long-term maintenance of an active lifestyle.

### Strengths and limitations

A strength of this study was the longitudinal study design with data collection at multiple time points (6-months (during), 12-months (post-intervention) and 24-months (12-months post-intervention) which provided in-depth accounts of participant experiences and a dynamic narrative describing how the motivational processes involved in adoption and maintenance of an active lifestyle evolve over a 24-month period [36]. Furthermore, this study is one of few longitudinal qualitative studies of older adults’ experiences of a PA intervention shown to be effective and cost-effective [42, 47, 48, 74–76]. The stratified purposive sampling strategy allowed for selection of REACT participants across a wide range of characteristics, providing rich data grounded in real-world experiences.

Although thematic saturation was reached during analysis, we cannot be sure that the addition of data from the participants who declined a follow-up interview would not have impacted the findings, and accept this as a common limitation of longitudinal qualitative studies [77]. Furthermore, recruitment of REACT participants with higher mobility limitations was challenging. Drawing on their perspectives would have provided a more holistic understanding how these older adults engage with PA, the barriers they face and how they can be better supported by session leaders. This knowledge will be important to further tailor successful programmes to the needs of older adults who experience more severe physical limitations.

## Conclusions

Our findings suggest that need-supportive environments that foster feelings of autonomy, competence and relatedness promote adherence to a structured, group-based exercise and behavioural maintenance programme. A transition from extrinsic motivation to intrinsic motivation and internalisation of feelings of competence (which were further strengthened by experiences of physical, mental and social benefits) were important for continuing engagement with the REACT programme and maintenance of an active lifestyle post-intervention. PA interventions for older adults should incorporate activities that are enjoyable and need-supportive; (a) making exercise social and enjoyable, (b) understanding participants’ capabilities and tailoring the programme accordingly, (c) capitalising on group support to motivate participants to try other activities and prepare sustainable active living plans. Further longitudinal qualitative research will allow us to understand how these (and other) processes impact engagement with a wider range of community-based activities, particularly focussing on older people with compromised mobility and older people who experience loneliness and/or social isolation.

## Supplementary Information

**Additional file 1. **REACT Participant Consent Form.

**Additional file 2. **Interview Topic Guides (6, 12 and 24 months).

**Additional file 3.** COnsolidated criteria for REporting Qualitativeresearch (COREQ) checklist.

## Data Availability

Interview transcripts can be made available on request, however the audio recordings of interviews cannot be shared publicly, as they are not anonymised. Participants may refer to people or places that could lead to their identification, or identification of the intervention provider.

## Abbreviations

REACT: Retirement in ACTion
PA: Physical Activity
SDT: Self-determination Theory
SPPB: Short Performance Physical Battery
COREQ: COnsolidated criteria for REporting Qualitative research
LOT: Lower Order Theme
