# Supplementary material for: A qualitative longitudinal study of motivation in the REtirement in ACTion (REACT) physical activity intervention for older adults with mobility limitations
Source: Int J Behav Nutr Phys Act. 2023 Apr 26;20:50. doi: 10.1186/s12966-023-01434-0 (PMC10131311; doi:10.1186/s12966-023-01434-0)
Supplement: Supplementary file 1 — Additional file 1. REACT Participant Consent Form. [file 12966_2023_1434_MOESM1_ESM.docx]

## Additional File 1. REACT Participant consent form

**REACT: REtirement in ACTion**

Centre: Participant number:

Dr A Stathi

Principle Investigator

Participant Consent Form

Please look at each of the statements and decide whether or not you agree. If you agree please sign your initials in the box, if you don’t agree leave the box blank.

| I confirm that I have read and understood the contents of the **REACT: REtirement in ACTion** Participant Information Sheet dated 23/03/2017 (Version 4) and have had the opportunity to consider the information, ask questions and have received satisfactory answers |  |
| --- | --- |
| I agree to the audio-taping of a face-to-face meeting between me and a REACT activity session leader |  |
| I agree to participate in one focus group (a small group where we will discuss the REACT project) if asked to do so, and to the audio-taping of any focus group that I take part in |  |
| I agree to the audio-taping of some of the physical activity or social and educational sessions I attend |  |
| I understand that the information collected about me will be stored on a computer and that it will be anonymised with a numeric code, which means I cannot be identified |  |
| I agree to my GP being informed of my participation in the study, and to my GP being contacted if the research team become concerned about my health or well-being |  |
| I consent to be contacted to discuss taking part in long-term follow up (up to 10 years) after the end of the study |  |
| I understand that relevant sections of my medical notes and data collected during the study, may be looked at by individuals from the Universities of Bath, Birmingham, Exeter or Oxford and from regulatory authorities, where it is relevant to my taking part in this research. I give permission for these individuals to have access to my records |  |
| I consent to my data being stored for use in future ethically approved research. |  |
| I understand that taking part is voluntary and that I can change my mind, withdraw from **any part** of the study, at **any** time without giving any reason and without penalty and without my medical care or legal rights being affected. |  |
| I understand that something I say and the results of the measures might be used in a written report but my name will not be used |  |
| I agree to take part in the above study |  |

| Name of participant |  | Date |  | Participant’s signature |
| --- | --- | --- | --- | --- |
| Name of person taking consent |  | Date |  | Signature |

Many thanks for your help


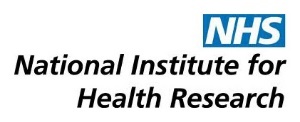
The REACT study Participant Consent Form V4 23/03/17 (IRAS No 169691)
