# Supplementary material for: A qualitative longitudinal study of motivation in the REtirement in ACTion (REACT) physical activity intervention for older adults with mobility limitations
Source: Int J Behav Nutr Phys Act. 2023 Apr 26;20:50. doi: 10.1186/s12966-023-01434-0 (PMC10131311; doi:10.1186/s12966-023-01434-0)
Supplement: Supplementary file 2 — Additional file 2. Interview Topic Guides (6, 12 and 24 months). [file 12966_2023_1434_MOESM2_ESM.docx]

**Additional File 2. Interview Topic Guides**

**Interview Topic Guide - Six Month Interviews**


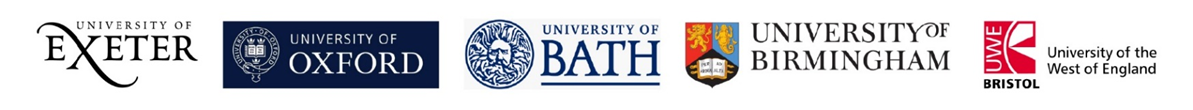


**REACT Topic Guide: Six-month Interviews**

**Interview Script**

Thank you very much for agreeing to speak to me about participating in the REACT study, we really appreciate your time.

Can I just check that you remember a researcher speaking to you on the phone to ask if you would like to take part in the project? After the researcher spoke to you, you said that you were happy to take part in the project, including being interviewed as part of the research we are carrying out. The reasons for this meeting is to find out why you wanted to take part in REACT and how things have been during the first six months of your involvement with this programme.

The interview will take around 45 minutes and will be recorded to ensure that we do not miss anything. When we start the interview I will ask you to give your name, so that we have a record of your agreement to take part. However, we will not use your name in any of our reports. If we use any quotes from you we will not give your name but use a false name.

Before we begin do you have any questions about doing the interview?

OK so the recorder is now going on….

| **Research Focus** | **Topic Guide Questions** |
| --- | --- |
| Introduction | **Firstly, are you happy for me to use this name? Or is there another which you usually go by?**  **Are you willing to agree to be interviewed by me? Thank you.**  I will now go into some questions about your interest in the REACT project. |
| Participant Information Sheet  Researcher Call  Benefits of being involved in research  One-to-One with Session Leader | - **Thinking back to when we first invited you to take part in the study, what did you think of the information document that we sent you to describe the REACT study?**   Prompt: Was there anything good about it? Was there anything off putting about it?  What influence your decision to take part?   - **What did you hope to gain from being involved in REACT in the first six months?**   Prompt: expectations, goals, personal health gain   - **What did you think about the call from the researcher who phoned about taking part in the project?**   Prompt: Was there anything good about it? Was there anything off putting about it?   - **What appealed to you about REACT?**   Prompt: GP invited you to take part, programme addressing your worries about limitations in mobility. Anticipated health benefits. Being involved in a group exercise class; meeting with people of your age living locally.   - **Other than personal benefit, do you think your involvement in the project is useful in other ways?**   Prompt: Benefits to other Older Adults, altruistic value of being involved in research.  **Moving on to your experiences of the first 6 months of the REACT programme**   - **How was the interaction with the session leader?**   Prompt: Were they professional, warm, empathetic, thoughtful, motivational? I*n what way? How did they make you feel? How did they support you? How did they communicate?*   - **At the end of the first face-to-face session did you have any remaining doubts about the programme? If so what were these?**   Prompt: What was your understanding of the REACT programme and what it involved? Understanding of the programme structure, need for commitment for 2 years of assessments, combination of exercise and social educational sessions. |
| Factors associated with REACT intervention effectiveness, REACT attendance and adherence to a daily PA. | **1.1 How did you find the exercise sessions?**  Prompt for: How did you find the exercise intensity?  How did you find the rate of progression? How did you find the comfort of doing the exercise with ankle weights? How did you find the balance exercises? How did you find the walking component?  **1.2 How did you find the social elements of the session (the non-exercise bits)?**  Prompt: How did you find the organisation of sessions, content of sessions, suitability of venue.  How did you get on as a group?  Did you experience any problems or concerns related to these sessions?  Is there anything we could do differently or better in these sessions?  Burden of attendance overall.  **1.3 After the first 12 weeks, REACT continued with one exercise session every week and one social/education session.**  **How was it for you making the change from two sessions to one session?**  Prompt for: Preference for one or two sessions a week.  Impact on motivation. Any involvement with other initiatives.  Plans to attend other initiatives with REACT group members.  Did you feel supported to make this change? Perceived level of support by REACT leader/partner to seek other local community initiatives  Did you have any difficulties starting up a new activity that you could do outside of REACT?  What got in your way (or what helped you to do this)?  Did you manage to do it in the end?  **1.4 What are you experiences of the Ambassador’s program?**  Prompt: Are you an ambassador? Do you know what a REACT Ambassador does?  **1.5 Were there benefits or downsides to having an ambassador in your REACT group?**  Prompt: What did you do as an ambassador? What was this experience like for you? Did they help in anyway during/outside sessions? |
| Participant experiences of barriers and facilitators to participation in the REACT intervention and daily PA | **2.1 Can you think of reasons that some people might not want to stay involved in the REACT study once they have started?**  Prompt: They did not think they would benefiting from the study?  Time required to exercise?  Time required to complete questionnaires?  Other commitments?  Interest in exercise?  Transport or venue issues?  **2.2 Were these problems that you faced?**  Prompt: How did this make you feel?  **2.3 So that we can learn from your experience, did you overcome them?**  Prompt for: How they did this? How would you encourage others to overcome these problems?  **2.4 If relevant did the REACT programme support you in overcoming the problems you faced?**  Prompt: one-to-one instructor advice, social session content, group help. |
| Experience of PA in day-to-day life | **3.1 Do you feel like you currently have an active lifestyle?**  Prompt: What does this involve? (Walking, gardening brining in the shopping, cleaning etc)  How has this changed since taking part in the REACT project?  **3.2 Do you feel like the REACT programme has played a role in this? If so can you explain how?**  Prompt: Encouragement, motivation, help to set goals, social activities, games, improved mobility, improved balance, and improved health. |
| Contextual factors, REACT attendance and daily PA | **4.1 What motivated you to want to be active before your involvement in REACT?**  **What motivates you to be physically active now?**  Prompt: Necessity, health enjoyment.  Where does the motivation originate? You, Friends, family, REACT friends, REACT instructor.  Does the REACT project play a role in your motivation to be physically active?  Do you see any differences in your views about physical activity?  **4.2 When you first started REACT you described your health as being (refer to Questionnaire data). Did this influenced your REACT attendance in anyway?**  Prompt: Did you experience pain and discomfort? How did this affect you? (if relevant) how did you overcome this barrier?  Did the REACT program help or support you in anyway? (Encouragement, advise, shared experiences). |
| Impact of theorised mechanisms (autonomy, relatedness, competence, modelling, and self-regulation techniques) depicted in the REACT Logic model on REACT attendance and daily PA | **5.1 What do you think influences or impacts on your decision to be active day-to-day?**  Prompt: What has kept you going? How do other people fit into this picture? How might they affect your decision to be active on any particular day?  **5.2 How do you feel about exercising as part of a group of people?**  Prompt: Why (enjoyment, helpful, social)  Has this included forming friendships within the REACT group?  Was this easy/ difficult to do? Encouraged by the facilitator?  **5.3 Are you confident taking part in REACT PA sessions?**  Prompt: What makes you feel confident or not?  Has this changed since starting REACT?  Did a lack of confidence stop you being physically active in the past?  **5.4 You said at the start of the programme that you were confident/not very confident (refer questionnaire data) in taking part in the REACT sessions or physical activity outside of the REACT sessions. How are things now?**  Prompt: What makes you feel confident?  Has this change since starting REACT?  **5.5 Did you set activity goals each week in your sessions?**  **5.6 What do you think about setting activity goals and keeping track of them?**  Prompt: How did you feel about this? Was it useful?  Do you feel any different in yourself for having taken part in the REACT? |
| Final Thoughts | - **Is there anything else that you would like to say about your experience in the first 6 months of the REACT programme?** |
| Closing Summary | Thank you very much for taking part in this interview, it’s been very helpful to hear about your views of the study. I really appreciate the time that you have given me today.  We will contact you again at the end of the 12 month REACT programme.  This will help us get a full picture of your experience of being a REACT member.  We will not be using your name in any of the reports that we write.  Thanks again. |

**Interview Topic Guide – 12-Month Interviews**

**
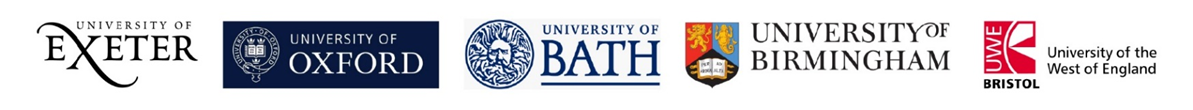
**

**REACT Topic Guide: 12-month Interviews**

**Interview Script**

Thank you very much for agreeing to speak to me about participating in the REACT study, we really appreciate your time.

The reasons for this meeting is to discuss your experience of being a REACT member for the last 12 months. Now that the programme has been completed it is a great opportunity to get your feedback about what worked well and how we can improve the programme further.

The interview will take around 45 minutes and will be recorded to ensure that we do not miss anything. When we start the interview I will ask you to give your name, so that we have a record of your agreement to take part. However, we will not use your name in any of our reports. If we use any quotes from you we will not give your name but use a false name.

Before we begin do you have any questions about doing the interview?

OK so the recorder is now going on….

| **Research Focus** | **Topic Guide Questions**  **12 Month Interviews** |
| --- | --- |
| **Introduction** | **Firstly, are you happy for me to use this name? Or is there another which you usually go by?**  **Are you willing to agree to be interviewed by me? Thank you.**  I will now go into some questions about your interest in the REACT project. |
| Factors associated with REACT intervention **effectiveness, REACT attendance** and adherence to **a daily PA.** | **Moving on to your experiences of the last 6 months of the REACT programme**  **1.1 After the first 6 months you said you had/ had not benefited from the REACT programme, is this still the case or has anything changed?**  Prompt: Walking, balance, strength, PA levels, social, well-being, confidence to be active, motivation to be active, general health  **1.2 If there is a change, why do you think this has changed?**  Prompt: attendance, experiencing barriers, time, illness, support/encouragement/ motivation form REACT instructor, group or family and friends.  **1.3 Do you feel any different in yourself for having taken part in the REACT programme?**  Prompt: Physical health, mobility balance, energy levels? Wellbeing, social life   - 1. **In the last 6 months how was the interaction with the session leader?**   Prompt: Were they professional, warm, empathetic, thoughtful, motivational? In what way? How did they make you feel? How did they support you? How did they communicate? Has it changed compared to the first 6 months? Improved, degraded, closer, friendlier  **1.5 How have you found the exercise sessions in the last 6 months?**  Prompt: How did you find the exercise intensity?  How did you find the rate of progression? How did you find the balance exercises? How did you find the walking component? Do you feel like you have progressed or improved these things?   - 1. **How has it been exercising as part of a group in the last 6 months, as you moved towards the end of the programme?**   Prompt: Why (enjoyment, helpful, social)? Is it something you miss or valued or have you since joined another group exercise class?   - 1. **How have you found the social education elements of the sessions in the last 6 months?**   Prompt: Fun, engaging, informative? How did you find the content of the sessions?   - 1. **How was it making the change from a SE session each week to one session per month?**   Prompt for: Preference for one session a week or one session a month. Impact on motivation.  Plans to keep up social activities with the group outside of REACT.  Did you feel supported to make this change? Perceived level of support by REACT leader/partner to seek other local community initiatives   - 1. **What did you take away from these sessions?**   Prompt: Were they useful, have you used them in your day to day life? Have they helped in maintaining an active lifestyle? In what way? If not, what would have helped?   - 1. **Was the Ambassador’s programme delivered in your group? If yes, what did you think of it? Were you interested in participating? What else could we do to support more people becoming Ambassadors?**   Prompt: what did you think about it? Were you interested in participating? What else can we do to support more people becoming ambassadors? Are you an ambassador? Do you know what a REACT Ambassador does? Benefits? Downsides? Did they help in and out of REACT sessions   - 1. **Since our last meeting (6 months ago in most cases) have you joined any other initiatives? If not do you have plans to?**   Prompts: What were your reasons for doing so? How did you hear about them? Were you encouraged to attend by REACT instructors, REACT friends or family members |
| Participant experiences of **barriers and facilitators** to participation in the REACT intervention and daily PA | - 1. **How often did you attend the REACT sessions (get attendance registers)**   Prompt: Every week, 2-3 times a month  **2.2 In the last 6 months did you face any barriers or anything that made you not want to or stopped you taking part in the REACT programme ?**  Prompt: They did not think they would benefiting from the study?  Time required to exercise?  Time required to complete questionnaires?  Other commitments?  Interest or motivation to take part in REACT exercise?  Transport or venue issues?  **2.3 So that we can learn from your experiences, did you overcome them?**  Prompt: How did you do this? How would you encourage others to overcome these barriers? Support from REACT instructor, REACT group, family, friends? Was anything you learnt in REACT helpful in overcoming these barriers  **2.4 If relevant did the REACT programme support you in overcoming the barriers you faced?**  Prompt: one-to-one instructor advice, social session content, group help.  **2.5 What was it about the REACT programme that kept you attending?**  Prompt: Enjoyment, social interaction, social network  Health benefits, improved mobility or balance  Interaction with session leaders  Habit formation  Social education sessions as a motive  Ease of getting to the session  Feeling valued and respected for taking part in the project  Contribution to the research  Support by family, friends, GP and others  **2.6 Can you think of any reasons people might not have attended REACT sessions over the last 6 months?**  Prompt: Didn’t see improvements? Not enjoyable or interested? Didn’t feel connected to the other REACT members? Time required to exercise? Other commitments? Transport issues? Lack of confidence in ability to take part? |
| Experience of PA in day-to-day life  Delivery of BCTs in REACT social education sessions | **3.1 At your 6 month interview you said that you did have/ did not have an active lifestyle? Is this still the case?**  Prompt: Have your physical activity levels change? What does this involve?  **3.2 If relevant do you feel like the REACT programme has played a role in this? If so explain how?**  Prompt: Encouragement, motivation, help to set goals, social activities, games, improved mobility, improved balance and improved health  **3.3 What sort of topics did you discuss in the social education sessions? (delivery)**  Prompt: Goal setting, action planning, overcoming barriers, managing setbacks to physical activity, monitoring your physical activity levels, benefits to physical activity  **3.4 What did you find useful, or take home from those discussions? (receipt)**  Prompt: ways to set goals and monitor your physical activity?  **3.5 Have you used any of these lessons day to day to maintain your physical activity levels? (enactment)**  Prompt: keeping an activity diary? Pedometer? Planning your upcoming events/clashes with your physical activities? |
| Contextual factors, REACT attendance and daily PA | **4.1 What motivates you to want to be physically active now?**  Prompt: Necessity, health, enjoyment.  Where does the motivation originate? You, REACT group, REACT instructor, friends, family?  Does the REACT project play a role in you motivations to be physically active?  **4.2 Before REACT what were your thoughts towards physical activity?**  Prompt: Positive, negative, fun, boring, necessary, social opportunity?  **4.3 In your 6 month interview you described your health as being (refer to interview data). Did this influence your REACT attendance in the last 6 months in any way?**  Prompt: Did you experience any pain and discomfort? How did this affect you (if relevant)? How did you overcome this barrier? Did the REACT programme help or support you in any way? (encouragement advice, shared experience) |
| Impact of theorised mechanisms (autonomy, relatedness, competence, modelling, and self-regulation techniques) depicted in the REACT Logic model on REACT attendance and daily PA | **5.1 Now that REACT has finished what do you think influences on your decision to want to be active day-to-day?**  Prompt: What motivates you to keep going? How do other people fit into this picture? How might they affect your decision to be active on any particular day?  **5.2 In the 6 month interviews you said that you were confident/not confident (check interview data) in taking part in the REACT sessions the REACT sessions. Did your confidence change over the last 6 months?**  Prompt: Increase, decrease? Reasons? Did REACT play a role? Did friends family, REACT groups, play a role?  **5.3 Are you confident in your ability to take part in PA now that you have completed the REACT programme?**  Prompt: How would you rate your confidence (1 to 10)? Why did you pick that? What makes you feel confident or not? How could we have helped you improve that score? Was there anything about the programme that was a barrier to you being confident? Functional limitations?  Did REACT play a role? Did friends family, REACT groups, play a role? Is your confidence limited or improved when you consider transport availability to activities? Is your confidence limited or improved when you consider support from family friends or REACT members?  **5.4 In the last 6 months did you set activity goals, start a new hobby or initiative or make plans about what you would do once REACT finished?**  Prompt: Discussions with session leader about your goals, what you wanted to achieve after REACT? Did you speak about other PA options in the area, ways to be physically active? Discussions with other REACT members about classes available in the local area, did you make plans with other members to attend them?  **5.5 If you didn’t discuss your ideas or plans for getting involved with other programmes/initiatives with REACT session leaders or members do you think it would have been useful to do so before REACT finished?**  Prompt: Would it have given your more options of activities in the local area, would you be more likely to make plans for physical activity? |
| Reasons for engaging in REACT/ Benefits of being involved REACT | **6.1 What did you hope to gain from being involved in the REACT programme?**  Prompt: expectations, goals, personal health gains, social  **6.2 Do you feel like you have achieved that?**  Prompt: expectations, goals, personal health gains, social |
| Final Thoughts | **7.1 If REACT sessions were available for one more year but for a small fee would you be interested in continuing to participate?**  Prompt: Can you explain why? Would you recommend it to other friends and family?  **Is there anything else that you would like to say about your experience of the REACT programme** |
| Closing Summary | Thank you very much for taking part in this interview, it’s been very helpful to hear about your views of the study. I really appreciate the time that you have given me today.  We will contact you again at the end of the 24 month REACT programme.  This will help us get a full picture of your experience of being a REACT member.  We will not be using your name in any of the reports that we write.  Thanks again. |

**Interview Topic Guide – 24-Month Interviews**

**
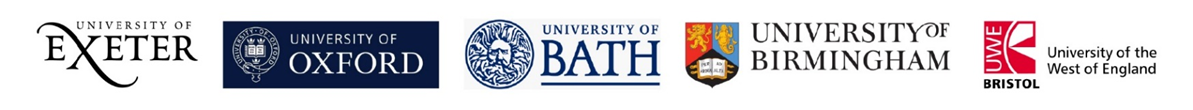
**

**REACT Topic Guide: 24-month Interviews**

**Interview Script**

Thank you very much for agreeing to speak to me about your participation in the REACT study, we really appreciate your time. The reason for this meeting is to discuss how things have been for you the year after the REACT exercise programme was completed. Remember, the aim of REACT was to support people to maintain an active lifestyle. A year later, it is now a great opportunity to get your feedback on how things have been for you.

The interview will take around 45 minutes and will be recorded to ensure that we do not miss anything. When we start the interview, I will ask you to give your name and today’s date, so that we have a record of your agreement to take part. However, we will not use your name in any of our reports. If we use any quotes from you, we will not give your name but use a false name.

Before we begin do you have any questions about doing the interview?

OK so the recorder is now going on….

| **Research Focus** | **Topic Guide Questions**  **24 Month Interviews** |
| --- | --- |
| **Introduction** | First, can you give me your full name please?  And todays date is……….  Are you willing to be interviewed by me? Thank you.  I will now go into some questions about your involvement in activities since the completion of the REACT sessions. |
| **Exercise Attendance** | **Since REACT finished have you attended any other exercise classes?**  **Prompt:** What type of classes? How often? Did you attend classes provided by the REACT partner or sessions provided by other community providers?  If no, then prompt: What were the reasons for not attending other exercise classes?  Explore barriers including motivational, personal, social, environmental barriers. |
| **Physical activity Participation** | **Since REACT finished, have you kept up your physical activity in any other ways?”.**  **Prompts**: have you kept doing (a) any exercises at home; (b) kept active by walking or other outdoor activities; (c) joined any kind of group or club that involves being physically active? If yes to any then prompt further about how often, whether with company, what motivated/enabled them to continue with these activities.  If c) is yes, is this club run by a REACT partner or are they run by other organisations? When no to any of the above, then prompt: What were the reasons for not keeping up physical activity??  Explore barriers including motivational, personal, social, environmental Also explore lapses (times that they stopped being active and continued after a time period). What were the reasons for those lapses? How did they manage to get back to physical activity? What kind of support was available and what facilitated their return to active lifestyle? |
| **Experiences of REACT Ambassador** | **Did you have any REACT ambassador in your group?**  If yes, did you get any support by the REACT Ambassadors during the REACT programme? If yes, then prompt: - What was that support? Was it useful? - Did you interact with the REACT Ambassador after the completion of the REACT programme in any way?  If no, then explore reasons that they did not interact with REACT Ambassadors. (There were no obligations by ambassadors to support people after the end of the intervention so here we only want to know whether Ambassadors where available during the programme whether they kept this relationship and continued informally to meet and socialise providing support.) |
| **Engagement with REACT peers** | **Have you kept contact with your REACT group members?**  If yes, how often do you meet? Do you organise days out/meeting for meals or coffee/going to cinema or other activities? |
| **Support for physical activity maintenance** | **In your effort to maintain being active, did you get any support by resources available in your community such as health visitors, health friends schemes, etc.?**  If yes, explore how useful that was? If not, what resources do you think would be helpful for you to maintain being active? |
| **Enjoyment of REACT** | Thinking of your involvement with the REACT study what: A. Were the things you enjoyed most? B. Were the things you least enjoyed/did not like? |
| **Reasons for engaging in REACT** | **Looking back at the reasons for joining REACT, what were the things you were expected to gain by being involved in REACT?**  **Prompt:** - expectations - goals - personal health gain If not, why do you think you did not get the gains you had expected?  If yes, would you say that you maintained some of these gains in the last 12 months since the end of the REACT programme?  Prompt for examples of gains they have maintained. If no, what got in the way? Use prompts to explore barriers –personal/social/other? |
| **Perceptions on how to improve REACT** | **How could we improve REACT to support people to maintain an active lifestyle after the end of the programme at 12 months in the future?**  **Prompts** ie.Transport issues |
| **Closing Summary** | Thank you very much for taking part in this interview and for all your support and input during the REACT study.  Your information is invaluable. I really appreciate the time that you have given me today and in our previous discussions.  I would like to wish you all the best. You will get more REACT newsletters with information about the outcomes of the study.  We will not be using your name in any of the reports that we write. Thank them again for their time |

.

**Topic Guide Development**

These semi-structured topic guides were developed and piloted with the REACT service user advisory group prior to use. This process indicated some issues with wording, questions that were slightly ambiguous in the participant topic guide, and questions not previously considered.

For instance, one question *“After the first 12 weeks, REACT continued with one exercise session every week and one social/education session. How did you find that?”* was edited to highlight the transition from two sessions to one session: *“After the first 12 weeks, REACT continued with one exercise session every week and one social/ education session. How was it for you making the change from two sessions to one session?”*
